# Short-Term Effect of Kinesio Taping of Lower-Leg Proprioceptive Neuromuscular Facilitation Pattern on Gait Parameter and Dynamic Balance in Chronic Stroke with Foot Drop

**DOI:** 10.3390/healthcare9030271

**Published:** 2021-03-03

**Authors:** Dongyun Lee, Youngsook Bae

**Affiliations:** 1 Department of Physical Therapy, Korea Su Medical Clinic, Bucheon 14643, Korea; east_shine@naver.com; 2 Department of Physical Therapy, College of Health Science, Gachon University, 191 Hambangmoe-ro, Yeonsu-gu, Incheon 21936, Korea

**Keywords:** dynamic balance, Kinesio tape, gait, proprioceptive neuromuscular facilitation pattern, stroke rehabilitation

## Abstract

The aim of this study is to identify the effectiveness of proprioceptive neuromuscular facilitation (PNF) leg Kinesio taping on gait parameters and dynamic balance in chronic stroke patients with foot drop. A total 22 chronic stroke patients were randomly assigned to experimental (n = 11) and control groups (n = 11). All subjects underwent conventional therapy and gait training for 50 min. The experimental group additionally received KT of tibialis anterior muscle (TA) and hamstring muscles according to the PNF pattern. The control group received KT of only TA. The primary outcome measures that the gait parameter are gait velocity, cadence, step length, and stride length. Dynamic balance was measured by the timed up-and-go test (TUG) time and activity-specific balance confidence scale (ABC) as the secondary outcomes. All of the measurements were performed baseline and 24 h after intervention. Our results showed that the experimental group showed significant improvements in gait velocity, cadence, step length, stride length and TUG, and ABC score compared with the control group. We conclude that the short term effect of application of lower-leg KT according to the PNF pattern increased the gait ability and dynamic balance of chronic stroke patients with foot drop.

## 1. Introduction

Stroke patients can distribute more than 80% of their body weight toward the non-paretic side, with only short durations of weight load on the paretic side, thereby reducing their balancing abilities [1,2]. This can limit the motion of stroke patients and increase their risk of falls [3]. Reduced gait ability in stroke patients is more problematic for the swing phase as opposed to the stance phase, and in particular, the gait in these patients is characterized by foot drop caused by reduced ankle dorsiflexion during the swing [4,5]. Foot drop is a pathology that may develop in hemiplegic patients as a result of inappropriate activation of the tibialis anterior muscle, a dorsiflexor of the ankle. Relevant interventions include functional electrical stimulation [6], ankle foot orthosis [7], and peroneal stimulation [8], which are primarily applied to the tibialis anterior muscle. Recently, Kinesio taping was found to improve static balance in stroke patients with foot drop [9]. Applying a Kinesio tape to the lower extremity during post-stroke rehabilitation is reported to relieve lower-extremity spasticity, improving lower–extremity motor function, improving balance, and enhance ambulation and gait parameters in patients [10].

Moreover, taping before proprioceptive neuromuscular facilitation intervention may promote functional improvement in stroke patients [11]. Proprioceptive neuromuscular facilitation (PNF) is a widely used intervention in the rehabilitation of patients with neurological and musculoskeletal disorders [12]. Recent studies investigated the effects of PNF on stroke patients [13,14], and some have reported that PNF improves gait in stroke patients [15,16]. The proprioceptive neuromuscular palpation pattern for the lower extremities consists of patterns D1 and D2. The starting position of D1 is hip extended, abducted, and internally rotated, ankle plantar flexed, foot everted, and toes flexed. Ending positions of the flexion pattern of D1 are hip abduction and internal rotation, knee flexion, and ankle dorsiflexion. The starting position of D2 is hip extended, adducted, and internally rotated, ankle plantar flexed, foot everted, and toes flexed. Ending positions of the flexion pattern of D2 are hip adduction, external rotation, knee flexion, and ankle dorsiflexion [17]. Two common features in the flexion patterns D1 and D2 are ankle dorsiflexion and knee flexion. In the principle of PNF, the elongated position of the pattern is a method of increasing the mobility of the muscle to facilitate muscle activation. KT also generally applied by stretching the tape in an elongated position [18]. In previous studies, KT was applied to TAM rather than functional muscle synergy to improve lower limb function [9]. However, there are insufficient studies to apply KT of the PNF pattern. In addition, the effect of applying KT to HAM and TAM, such as functional muscle synergy, is unclear.

The purpose of this study is to identify the effects of PNF leg Kinesio taping on gait parameters and balance confidence in stroke patients with foot drop. We hypothesized that applying the Kinesio tape to the TAM and HMs according to the PNF pattern would improve gait parameter and dynamic balance in stroke patient with foot drop more than when applied only to the TAM.

## 2. Materials and Methods 

### 2.1. Research Design

This study had a randomized controlled trial design. The blocking ensured equal numbers of participants in the experimental and control groups. Participants were randomly assigned to one of the two groups depending on the code shown in a sealed, opaque envelope. Randomization was performed by an investigator not related to the recruited participants. Simple randomization was performed using Windows Microsoft Excel (Microsoft Corporation, Redmond, WA, USA). Before the experimental process, the participants were informed in detail about the study procedure and safety, and they signed a written informed consent. All procedures were approved by Gachon University’s Institutional Review Board (clinical trial registration number: KCT0003677) were conducted in accordance with the 1975 Helsinki Declaration. The data for the study were collected from May 2017 to July 2017.

### 2.2. Participants and Procedure

Twenty-two patients affected by a stroke were enrolled, and they were hospitalized at rehabilitation center. All participants met the following criteria: (1) 12 months or more since the diagnosis of stroke with foot drop, (2) independent gait possible over 10 m without walking aid, (3) Modified Ashworth scale score of ≤2, (4) no surgical procedure performed on lower limbs, and (5) absence of other medical complications. Patients with an open wound that hinders the application of Kinesio tape, patients with skin symptoms after applying the tape, and those with pre-existing neurological disorders, progressive disease, Mini-Mental State Examination score below 24, and other concurrent medical conditions were excluded. 

To determine the sample size, G-Power 3.19 software (Heinrich Heine University Düsseldor, Düsseldorf, Germany) was used. To calculate the sample size, repeated measures analysis of variance within and between interactions was used, and the alpha error probability and power were set at 0.05 and 0.8, respectively. In addition, the effect size was set at 0.35; the number of groups and number of measurements were set as two and two, respectively [19]. The calculated sample size was 20% and 10% dropout rate was considered. Therefore, sample size was determined as 22 participants.

**Figure 1:**
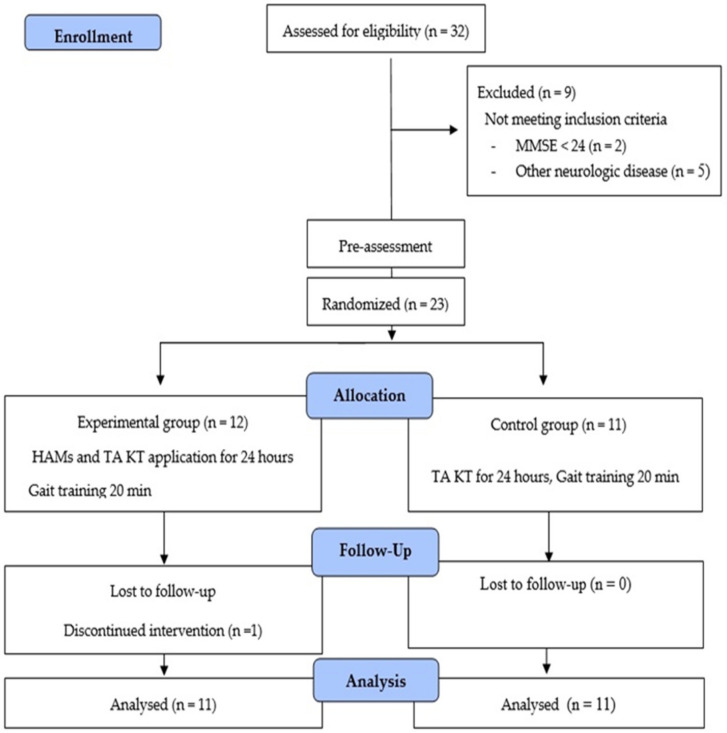
Flow chart of this study.

At beginning of this study, a total of 32 patients with chronic stroke were screened. Of these, nine were excluded for not meeting the inclusion criteria, resulting in a total of 23 participants. Baseline measurements were taken before random allocation. Participants were randomly assigned to one of the two groups depending on the code shown in a sealed, opaque envelope. Randomization was performed by an investigator not related to the recruited participants. Simple randomization was performed using Windows Microsoft Excel (Microsoft Corporation, Redmond, WA, USA). Participants were randomly divided in the experimental group (n = 12) and the control group (n = 11). However, one participant in the experimental group was excluded for not adhering to the study protocol; thus, a total of 22 participants, 11 in both the experimental and control group, were analyzed. Then, patients in both groups underwent conventional therapy (CT) for 30 min and gait training for 20 min on a treadmill after KT. For the experimental group, the Kinesio tape was applied on the TAM and HMs, whereas for the control group, it was applied only on the TAM (Figure 1). 

Before the experimental process, the patients were informed in detail about the study procedure and safety, and they signed a written informed consent. The general characteristics of the participants were measured before assessment. KT is usually used for three days, but if side effects, such as skin itching, occur, the tape should be removed immediately [20]. Previous studies assessed the short-term attachment rather than long-term attachment in the KT application [9,15]. Kinesio taping has the greatest increase in muscle activity after 24 h of application [21]. Therefore, in this study, measurements of gait ability and balance confidence were performed at baseline and after 24 h. 

### 2.3. Intervention (Applying Kinesiology Taping)

All patients underwent CT led by a physical therapist for 30 min. The treatment applied to the patient was based on the principle of neuromuscular developed therapy and a task-oriented approach. It consisted of functional training that included progression from simple movements to complex movement patterns such as torso and leg muscle training, posture control, and weight control. Loading and movement of the legs. Each session consisted of approximately 10 min to facilitate alignment of the lower limbs and 20 min of graded application of functional tasks such as sitting up or walking. We increased the number of iterations to facilitate progress within each task. Subsequently, walking training was performed on a rehabilitation treadmill (Hansin Sports, Seoul, South Korea) for 20 min. The initial speed on the treadmill was determined for each patient based on the mean walking speed in the 10-m walk test conducted before the intervention. For the first 2–3 min, the participants warmed up on the speed level half of that set for training. Then, they walked for 15 min at the set speed and then cooled off for 2–3 min. The therapist stayed next to the participant to adjust the treadmill speed and guide the participant to walk safely. Each participant were performed the CT and walking training by a physical therapist in charge of them, and there were six therapists performing interventions.

**Figure 2:**
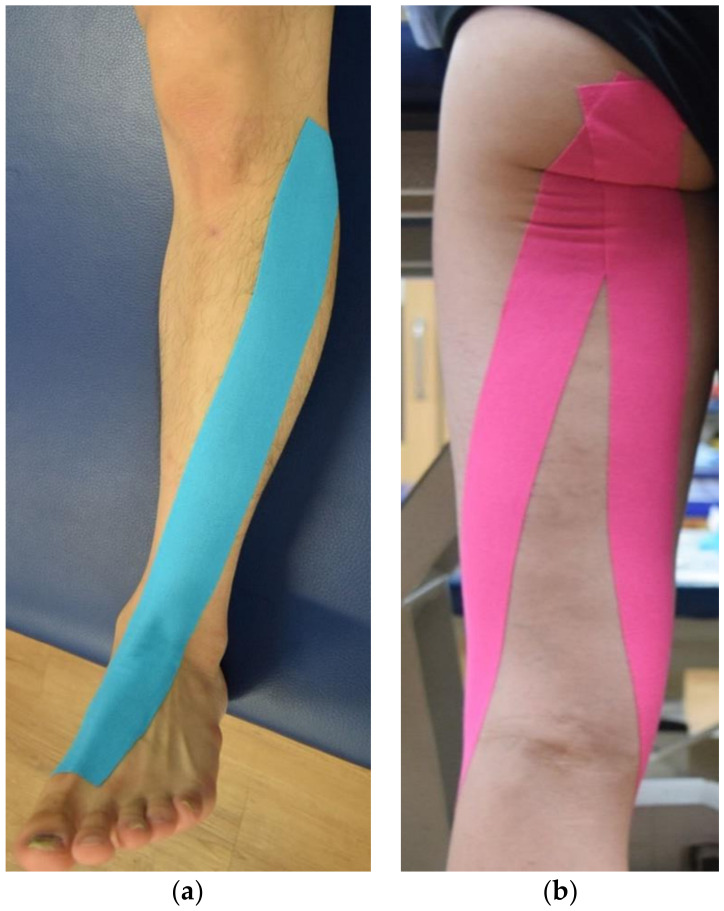
Application of kinesiology tape to the tibialis anterior muscle (**a**), hamstring muscles (**b**).

A 5-cm wide Kinesio^®^ Tex Tape (Kinesio Holding Corporation, Tokyo, Japan) was used, and taping was performed by one qualified physical therapist with more than five years of experience who was blinded to the purpose of the study. The tape was applied to the paretic leg before performing CT. It was applied to the TAM and HMs in the experimental group, whereas it was applied only to the TAM in the control group. The tape was applied to the lower extremity on the paretic side, and when necessary, patients were asked to remove the hair at the site of application after obtaining consent from them before the study. The therapist applied the tape from the lateral condyle of tibia to the base of first metatarsal bone with the participant in the supine position, after maximal plantar flexion of the ankle joint (Figure 2a) [22]. To apply the tape to the HMs, the table height was adjusted to the level of the participant’s anterior superior iliac spine while standing, and the participant flexed the trunk to induce anterior tilting of the pelvic bone. Following this, the tape was applied from the ischial tuberosity of the pelvis to the medial condyle of tibia and fibular head (Figure 2b) [23]. During application, the tape was not stretched for 5 cm from the initial site and was then stretched 30% for the remaining parts [18].

### 2.4. Outcome Measures

Gait ability was measured by the spatiotemporal parameters of gait as the primary outcome, and dynamic balance was measured by the timed up-and-go test (TUG) time and activity-specific balance confidence (ABC) scale as the secondary outcomes. The outcome measurements were performed by two physical therapists who did not performed intervention at the center.

Spatiotemporal parameters of gait were measured using the GAITRite System (GAITRite^®^ CIR System Inc., New York, NY, USA), a portable gait analysis tool connected via a serial port to a computer for the automated measurement of spatiotemporal gait parameters. GAITRite consists of an 810 × 89 × 0.625 cm (length × width × height) instrumented mat with 27,648 embedded pressure-sensitive sensors spaced at 1.27 cm and arranged in a 48 × 576 grid. The active area of the mat is 7.31-m long and 0.61-m wide. The sampling rate was set at 80 Hz. The obtained data were analyzed using a gait analysis software (GAITRite PLUS version 4.7, CIR System Inc., USA). Gait parameters were measured as per the published guidelines using the GAITRite system [24]. Participants wore their own footwear. Further, to maintain the gait speed on the mat, patients initiated and terminated walking a minimum of 3 m before and after the start and end points on the walkway, respectively. To ensure safety, an investigator walked alongside the participants. The measured parameters were velocity, cadence, step length, and stride length of the paretic side. The test-retest reliability of GAITRite system was good, with an intra-class correlation coefficient (ICC) of 0.98–0.99 [25]. 

TUG may assess fear of falling in stroke patients [26] and is a reliable measure to detect postural balance, mobility and lower limb movements in individuals with chronic stroke [27]. Participants sat on an armless chair, and upon the voice signal “start,” they walked 3 m along a line on the floor at a comfortable speed, walked back to the chair, and sat down in the chair. A total of three measurements were conducted and the mean value was used.

The ABC scale has been used with various populations, including older adults and stroke patients [28]. It measures one’s confidence level of walking without falling not only at home but also outside; thus, it can be used for individuals who retain a certain level of functional ability. In this study, we used the Korean version of the valid ABC scale [29]. The participants were instructed to walk within the rehabilitation center premises for more than 30 min, and a 16-item questionnaire rating their confidence from 0% (no confidence) to 100% (very confident) was implemented. The mean score was considered the total score for ABC, and a higher score indicated a higher level of balance confidence. The internal consistency reliability of ABC was 0.94 and test–retest reliability was good with ICC of 0.85 (95% CI, 0.68–0.93) [30].

### 2.5. Statistical Analysis

Statistical analysis was performed using SPSS version 24.0 software (IBM, Armonk, NY, USA) for Window 10. The normal distribution of data was determined by the Shapiro–Wilk test, and all outcome variables were normally distributed. In this study, the parameter test was used because normal distribution was seen. Independent t-test and χ^2^ test were performed to compare general characteristics and baseline between the groups. Group (2) × time (2) two-factor mixed ANOVA was performed to determine whether there was an interaction between the group and the time point. The effect sizes of the interaction effect were calculated as η^2^ to determine meaningful changes between groups. An effect size of up to 0.02, 0.13, and 0.26 indicated small, moderate, and large changes, respectively [31].

All values are expressed as mean ± SD, and *p* < 0.05 was considered statistically significant.

## 3. Results

Baseline characteristics of participants are included in Table 1. The primary finding of this study was that the experimental group showed a significant increase in the velocity (*p* = 0.013), cadence (*p* = 0.002), step length (*p* = 0.002), and stride length (*p* = 0.005), and secondary finding showed a significant increase in TUG (*p* = 0.007) and ABC (*p* = 0.001). The control group showed a significant increase of step length (*p* = 0.003) and ABC (*p* = 0.005). In between groups, experimental group significant increase in velocity (*p* = 0.029, η^2^ = 0.217), cadence (*p* = 0.019, η^2^ = 0.245), step length (*p* = 0.032, η^2^ = 0.210), and stride length (*p* = 0.023, η^2^ = 0.231), and TUG (*p* = 0.016, η^2^ = 0.257), ABC (*p* = 0.001, η^2^ = 0.432) than control group (Table 2).

## 4. Discussion

This study showed that the experimental group significantly increased in gait parameters of cadence and stride length, and ABC score compared with the control group. These findings confirm our research hypothesis that additional application of the KT according to PNF pattern before CT and gait training enhances gait function and dynamic balance.

Post-stroke gait patterns are characterized by inappropriate ankle dorsiflexion and knee flexion in the swing phase [32]. Inadequate knee flexion in swing phase has been associated with several causes [33]. Therefore, rehabilitation for patients with an extended walking pattern should focus on strengthening of the entire flexion pattern [34]. PNF patterns of the lower extremities reinforce muscular strength and enhance muscle re-education for lower extremity function such as gait [15]. A previous study has reported that KT enhances muscle activation and re-education by increasing the subcutaneous space, enhancing blood flow, and providing tactile stimulation [18]. Applying the Kinesio tape on the TAM in stroke patients significantly increases gait velocity and step length over four weeks [22]. In previous studies, gait velocity and step length were significantly increased when KT was applied for longer than 4 weeks [35], the gait velocity and number of steps were significantly changed when applied for less than 24 h [9]. 

In the present study, both the experimental and control groups had significant increases in step length. These results confirm previous findings that applying the Kinesio tape to the TAM is effective in improving the gait function in stroke patient. Furthermore, the experimental group showed a significantly improvements in step length compared with the control group. When gait velocity increases, stride length and cadence are significantly increased [36], and these can be used as indices for gait training. Higher gait velocity means that TAM and HM activity is increased in the swing phase [37]. In our finding, the experimental group showed a significantly greater increase in velocity, stride length and cadence than control group. In particular, longer stride length indicates that improved more paretic leg movement during the swing phase. Therefore, authors suggested that applying the KT to both TAM and HMs according to the PNF pattern, as opposed to applying the tape only to the TAM, would show better improvement in gait function by facilitating TAM and HM activity in the swing phase. Thus, it is believed that this result is obtained by increasing the efficiency of the KT according to PNF pattern by resolving the problem of inappropriate ankle dorsiflexion and knee flexion during the swing phase in foot drop. 

Our findings show that the TUG and ABC showed a significantly greater increase in experimental group. TUG measures dynamic balance; it has been shown to identify the risk of falling [38] and may better predict falls than gait speed in stroke patients [39]. Slower gait speed, shorter stride length, increased stride width, and prolonged double limb support time are found to be associated with a preexisting fear of falling, and gait function is related to gait confidence, such as fear of falling [40]. In our study, ABC scores significantly greater increase compared with the control group. This suggests that KT according to the PNF pattern most significantly improved dynamic balance because gait parameter was increased. However, positive effects of KT in enhancing balance function recovery were noted in a few studies, indicating that either a short-term or a long-term application of KT can make some difference [10]. Therefore, the authors suggested that long-term studies are needed to prove the effect of the balance of KT according to PNF pattern application.

### Limitation of the Study

This study provides information that the application of KT to the PNF pattern is effective in improving the gait and balance ability of stroke patients in clinical practice, compared with previous studies. However, this study has a few limitations. First, we only examined outcomes 24 h after the application of the Kinesio tape; thus, we could not identify long-term effects. We propose that a follow-up study of at least four weeks’ duration is required to confirm the long-term effects of PNF pattern KT. Second, there is a possibility of selection bias for patients with high functional states, as we included patients with a Modified Ashworth Scale score of ≤2, but we could not perform this study on patients with low functional ability. Finally, we compared a group of patients with Kinesio tape application only to the TAM with a group of patients with Kinesio tape application to both TAM and HMs according to the PNF pattern. A previous study reported about quadriceps muscle (QM) strengthening as a result of rehabilitation in patients with extended walking, but found no significant changes in gait and balance after additionally KT the QM before CT [41]. Therefore, we did not consider comparing the results of Kinesio tape application to the TAM and HMs with those of Kinesio tape application to the TAM and QM. To address these limitations, we plan to perform an additional study to compare the changes in gait parameters after applying the Kinesio tape to the TAM and HMs according to the PNF pattern with changes after applying the tape to the TAM and QM. We are also planning a long-term study. 

## 5. Conclusions

Our study showed that KT for TAM and HM resulted in a greater improvement in gait parameters and dynamic balance in stroke patients compared to applying KT only to TAM. Therefore, clinical rehabilitation programs that include PNF pattern KT along with conventional treatments can help improve gait and balance in stroke patients with foot drop.

## Figures and Tables

**Table 1:** Baseline characteristics of the participants.

	Experimental Group (n = 11)	Control Group (n = 11)	*p*-Value
Sex (male/female)	8/3	7/4	0.647 *
Diagnosis (infarction/hemorrhage)	7/4	3/8	0.19 *
Age (years)	46.3 ± 7.1	53.8 ± 12.8	0.474 ^†^
Onset time (months)	19.6 ± 13.7	23.2 ± 14.0	0.556 ^†^
Height (cm)	168.2 ± 9.5	162.9 ± 7.5	0.167 ^†^
Weight (kg)	68.5 ± 8.8	65.3 ± 10.0	0.431 ^†^
MMSE (score)	28.5 ± 2.3	27.4 ± 2.0	0.209 ^†^
MAS (score)	1.0 ± 0.8	1.5 ± 0.7	0.096 ^†^
Gait spatiotemporal parameter			
Velocity (m/s)	0.38 ± 0.37 69.50 ± 27.31 30.69 ± 13.14 61.95 ± 27.17	0.40 ± 0.25 69.84 ± 69.84 32.43 ± 15.46 59.75 ± 30.19	0.086 0.976 0.779 0.859
Cadence (step/min)	37.30 ± 29.73	34.14 ± 19.60	0.771
Step length (cm)	41.88 ± 19.40	44.77 ± 29.37	0.788

* Statistical analysis was performed using the chi-square test. ^†^ Statistical analysis was performed using the independent *t*-test. Values are expressed as mean ± standard deviation. MMSE: Mini-Mental State Examination; MAS: Modified Ashworth scale, ABC: Activities-specific balance confidence scale.

**Table 2:** Comparisons of gait ability and confidence variables between before and after intervention.

Variables	Experimental Group (n = 11)			Control Group (n = 11)			Group * Time		
	Baseline	24 h	*p*	Baseline	24 h	*p*	F (1, 21)	*p*	η^2^
Gait spatiotemporal parameter									
Velocity (m/s)	0.38 ± 0.37	0.50 ±0.48	0.013	0.40 ± 0.25	0.42 ± 0.23	0.079	5.541	0.029	0.217
Cadence (step/s)	69.50 ± 27.31	77.24 ± 27.17	0.002	69.84 ± 69.84	70.48 ± 28.94	0.768	6.477	0.019	0.245
Step length (cm)	30.69 ± 13.14	35.53 ± 15.73	0.002	32.43 ± 15.46	34.32 ± 15.98	0.003	5.312	0.032	0.210
Stride length (cm)	61.95 ± 27.17	69.83 ± 33.62	0.005	59.75 ± 30.19	61.69 ± 32.69	0.086	6.017	0.023	0.231
TUG (s)	37.30 ± 29.73	27.36 ± 20.66	0.007	34.14 ± 19.60	32.78 ± 21.10	0.380	6.905	0.016	0.257
ABC (score)	41.88 ± 19.40	68.52 ± 22.78	0.001	44.77 ± 29.37	54.09 ± 28.50	0.005	15.222	0.001	0.432

TUG: Time up-and-go test; ABC: Activities-specific balance confidence scale.

## Data Availability

The data presented in this study is provided upon request of the corresponding author. Data cannot be used publicly due to restrictions such as personal information.
